# The health effects of soy: A reference guide for health professionals

**DOI:** 10.3389/fnut.2022.970364

**Published:** 2022-08-11

**Authors:** Mark Messina, Alison Duncan, Virginia Messina, Heidi Lynch, Jessica Kiel, John W. Erdman

**Affiliations:** ^1^Soy Nutrition Institute Global, Washington, DC, United States; ^2^Department of Human Health and Nutritional Sciences, University of Guelph, Guelph, ON, Canada; ^3^Nutrition Matters, Inc, Pittsfield, MA, United States; ^4^Kinesiology Department, Point Loma Nazarene University, San Diego, CA, United States; ^5^Scientific and Clinical Affairs, Medifast Inc., Baltimore, MD, United States; ^6^Division of Nutritional Sciences and Beckman Institute, Department of Food Science and Human Nutrition, University of Illinois at Urbana/Champaign, Urbana, IL, United States

**Keywords:** soyfoods, counseling, patients, isoflavones, review, protein, recommendations, clients

## Abstract

Soy is a hotly debated and widely discussed topic in the field of nutrition. However, health practitioners may be ill-equipped to counsel clients and patients about the use of soyfoods because of the enormous, and often contradictory, amount of research that has been published over the past 30 years. As interest in plant-based diets increases, there will be increased pressure for practitioners to gain a working knowledge of this area. The purpose of this review is to provide concise literature summaries (400–500 words) along with a short perspective on the current state of knowledge of a wide range of topics related to soy, from the cholesterol-lowering effects of soy protein to the impact of isoflavones on breast cancer risk. In addition to the literature summaries, general background information on soyfoods, soy protein, and isoflavones is provided. This analysis can serve as a tool for health professionals to be used when discussing soyfoods with their clients and patients.

## Introduction

A substantial amount of soy-related research has been conducted over the past 2–3 decades, as about 2,000 papers are indexed in PubMed annually. Much of this research has been conducted because the soybean is a uniquely rich source of isoflavones (1). Isoflavones have been purported to exert several health benefits, from reducing cancer risk (2, 3) to alleviating menopausal symptoms (4) and improving memory (5). On the other hand, isoflavones are also routinely classified in the scientific literature as endocrine disruptors (6–16), despite the conclusions of a recently published comprehensive technical review that neither soy nor isoflavones warrant such classification (17). Consequently, even though soyfoods have been consumed for centuries by Asian populations (18) and have become increasingly mainstream outside of Asia, they are not without controversy.

In addition to the traditional Asian soyfoods, questions have been raised about the healthfulness of soy protein ingredients (concentrated sources of soy protein) because of the processing they undergo (19, 20) and about soybean oil, because of its high omega-6 (n-6) polyunsaturated fatty acid (PUFA) content (21–23). Soy protein ingredients are the base for manufacturing modern soy products such as soy-based meat alternatives and are widely used by the food industry for their functional properties (24). Both soy protein and soybean oil play a huge role in the US food supply (17, 25).

Between 1998 and 2009, many concerns about soy were raised (26–31) and challenges to the proposed health benefits were made (32–34). These developments led to lay publications claiming soy is detrimental to health (35–37). This may explain why, according to a 2021 survey of 1,500 US consumers, fewer than half of baby boomers consider soyfoods to be somewhat/very healthy compared to 68% of generation Z-ers, as the latter demographic was not exposed to this information as adults (38). Hesitancy about soy exists despite worldwide recommendations emphasizing the personal and planetary health benefits of plant-based diets (39, 40).

Many health professionals may not be in a position to accurately advise their clients and patients about soy because of the vast amount of research that has to be reviewed in order to gain a working understanding of the subject. This review will provide concise literature summaries (400–500 words) along with a short perspective on the current state of knowledge of the more hotly debated and widely discussed soy-related topics, so that health practitioners will be able to provide well-informed recommendations and counsel. Prior to presenting these summaries, general background information on soyfoods, soy protein, and isoflavones is provided.

Regarding the perspectives, because there is some subjectivity involved in the evaluation of the literature when conducting narrative reviews, a conservative approach has been adopted when reaching conclusions about the strength of the data. As much as possible, emphasis was placed on systematic reviews, meta-analyses, and the positions of independent health agencies when formulating the perspectives. Generally speaking, clinical trials influenced conclusions more than observational studies, but some nuance is still required. Small, short-term clinical trials evaluating markers of disease risk may carry less weight than large, prospective, observational studies with long follow-ups evaluating disease outcome.

## Soyfoods overview

### Traditional Asian soyfoods

Historical records suggest the use of soybeans as a food originated in China possibly around 2,000 years ago (41), although archeological evidence indicates soybean domestication may have occurred several thousand years earlier (42). From China the soybean spread to Japan and other Southeast Asian countries although recent data suggest there may well have been multiple independent efforts to domesticate wild soybeans (43). There are two general categories of Asian soyfoods, fermented and unfermented. Fermented soyfoods include natto, tempeh and miso whereas unfermented foods include soymilk and tofu (Table 1). Tempeh is a more recent creation having been developed in Indonesia around the 1600s (44). Globally, most soy is consumed in the unfermented form (excluding soy sauce, which is a condiment, not a food) (45).

**Table 1:** Commonly consumed traditional soyfoods.

Soybeans A species of legume *(Glycine max)* native to East Asia, widely grown for its edible bean. It is classified as an oil seed not a pulse because of its high fat content. Edamame Soybeans in the pod which are harvested at about 80% maturity, so they retain more water and sugars. The pods are boiled or steamed and may be served with salt or other condiments. Tofu (soybean curd) Prepared by coagulating soymilk and then pressing the resulting curds into solid white blocks of varying softness; it can be silken, soft, firm, extra firm or super firm. The three main commonly used categories of coagulants are salts (e.g., calcium sulfate, magnesium chloride and calcium chloride), acids (e.g., glucono delta-lactone), and enzymes (e.g., papain and proteases). Soymilk Made by soaking and grinding soybeans, boiling the mixture, and filtering out remaining particulates. It is a stable emulsion of oil, water, and protein. Its original form is an intermediate product of the manufacture of tofu. Miso Made by fermenting soybeans for a few days to over a year with salt and koji (the fungus Aspergillus oryzae) and sometimes rice, barley, seaweed, or other ingredients. Natto A traditional Japanese food made from soybeans that have been fermented with Bacillus subtilis var. natto. It is often served as a breakfast food along with karashi mustard, soy or tare sauce, and sometimes Japanese bunching onion. Tempeh A traditional Indonesian (Javanese) food made by a natural culturing and controlled fermentation process that binds soybeans into a cake form. A fungus, Rhizopus oligosporus, is used in the fermentation process and is also known as tempeh starter.

### Soy protein ingredients

There is a vast array of foods made using concentrated sources of soy protein, often referred to as soy protein ingredients, as a base. The starting point for these ingredients are soybean flakes, which are produced by crushing soybeans and removing the oil using a food grade solvent. The primary soy protein ingredients are soy protein isolate (SPI), soy protein concentrate (SPC) and soy flour, which are comprised of ≥90, 65–90, and 50–65% protein, respectively (46).

These ingredients have been extensively used by the food industry for decades. They are used in a wide range of foods because of their functional properties such as solubility, gelation, hydrating capacity, emulsification, adhesion/cohesion, and foaming (25, 47). Because they are added to foods in such small quantities when used in this way, their contribution to protein intake is negligible. More relevant from a nutritional perspective is the use of these products in the manufacture of dairy and meat alternatives, and as a means of delivering high quality protein in a variety products such as cereals, energy bars and infant formula (25).

### Soy protein quality

The protein digestibility corrected amino acid score (PDCAAS) is the method for determining protein quality accepted by most regulatory bodies including the Food and Agriculture Organization (FAO) of the United Nations and the US Food and Drug Administration (FDA). PDCAAS is determined by comparing the profile (mg/g protein) of indispensable amino acids (IAAs) in a protein with the biological requirement for the IAAs and then correcting for digestibility based on the digestibility of protein at the end of the large intestine (fecal digestibility) in rats.

Considerable soy protein quality research has been conducted, although until recently that research focused mostly on concentrated sources of soy protein such as SPI and SPC. Soy protein is well-digested and has an IAA pattern that closely matches human requirements. The limiting amino acids in soy protein are the sulfur-containing amino acids (SAA), methionine and cysteine. SPI and SPC have a PDCAAS of ~1.0 (scores are truncated at 1.0 or 100% using this methodology) (48–50). Research published in 2011 showed the PDCAASs for SPI and SPC are slightly higher than for beef (0.92) and much higher than for other plant proteins (e.g., pea protein concentrate, 0.73; kidney beans, 0.68; pinto beans, 0.63; rice, 0.53; wheat gluten, 0.25).

However, beginning in 2011, the FAO convened a series of meetings of experts in protein quality methodology. The reports of these meetings recommend gradually shifting from the PDCAAS to one of five potential methods for assessing protein quality. The most well-known is the digestible indispensable amino acid score (DIAAS), which has been widely used in the animal feed industry and has received the most support. Since some methodological issues remain to be resolved, and limited data exist on the quality of proteins using this method, it will likely be many years before regulatory bodies adopt the DIAAS (51). Nevertheless, it is now quite common to see in the literature protein quality data based on DIAAS.

In contrast to the PDCAAS, when using the DIAAS, protein quality scores are not truncated (i.e., scores can be above 1.0 or 100%), and digestibility is based on the digestibility of individual IAAs determined at the end of the small intestine (ileal digestibility) using pigs or humans. Additionally, the FAO developed three new IAA scoring patterns for evaluating protein quality (birth to 6 months; 6 months to 3 years and >3 years [which includes older child, adolescent, and adult]). Recently, tofu (52), soymilk (52), and a popular soy-based burger (53) received scores using the DIAAS of 97, 117, and 107%, respectively when using the IAA reference pattern for the older child, adolescent, and adult. In comparison, when using this same reference pattern, 80% lean ground beef and a popular pea protein-based burger received scores of 110 and 83%, respectively (53).

### Soy protein and muscle mass

There has been considerable investigation of the ability of soy protein to stimulate muscle protein synthesis (MPS) and to promote gains in muscle mass and strength in response to resistance exercise training (RET). Whey protein, which represents ~20% of the protein in cow's milk, has traditionally been considered the optimal protein for building lean body mass (54–56) because it is high in the branched chain amino acid leucine, an important trigger for MPS (57). Acute feeding studies (~4 h) show consuming whey protein stimulates MPS to a greater extent than soy protein (58–64). However, the entire hypertrophic period following resistance exercise is unlikely to be captured by short-term studies. Therefore, acute studies identifying differences among protein sources measuring MPS may not predict long-term changes in gains in muscle mass and strength (65, 66). The results of a recently published meta-analysis that included nine clinical trials supports this conclusion in that it was found soy protein promotes gains in muscle mass and strength similarly to whey and other animal proteins (67).

Based on their systematic review, meta-analysis and meta-regression, Morton et al. (68) concluded that protein source likely plays a minor, if any, role in determining RET-induced gains in fat-free mass and strength over a period of weeks. Recently, Morgan and colleagues (69) determined that protein quality likely has a significant, although small benefit in both young and older adults on indices of muscle protein anabolism. Their analysis found that higher protein quality was associated with superior strength gains in response to RET but not with changes in lean body mass (69). While there may be some uncertainty about the precise impact of protein quality, there is little disagreement that the protein requirements of individuals engaged in endurance and RET exceed those of the general population by anywhere from as little as an additional 50% to as high as 250% (68, 70–74).

### Soy protein and body weight management

There is considerable interest in the role of protein in body weight management (75, 76). Some evidence suggests that dietary protein is the most effective macronutrient at providing a satiating effect (77). After adjustments for a wide range of factors, Lieberman et al. (78) recently found that among 14 developed countries, protein intake, regardless of demographic and lifestyle factors, was consistently ~16% of total energy, which is nearly twice the amount needed to meet the adult protein recommended dietary allowance (RDA) (79). In contrast, there were relatively large variations in the amount of carbohydrate and fat consumed by country.

Lieberman and colleagues (78) proposed that protein intake is tightly regulated by biological control mechanisms. This proposal concurs with the protein leverage hypothesis (PLH). (80). According to the PLH, there is a strong biological propensity to regulate the quantity of protein consumed (81). According to this hypothesis, diets with a lower protein content as a percentage of calories, could stimulate caloric intake. However, some researchers have concluded that “… no individual nutrient is a friend or a foe when it comes to weight loss and its maintenance.” (82).

A review published in 2008 found that soy is as good as other protein sources for promoting weight loss (83). More recently, the authors of a double-blind, randomized cross-over study involving 17 healthy adults concluded that consuming soy protein exerts comparable effects to whey protein on appetite profile, energy metabolism, and subsequent energy intake. For this study, during each of the three testing visits, the participants were given one of three breakfast meals and an *ad libitum* lunch, while appetite ratings and metabolic testing were assessed for the following 3 h. Energy intake at lunch was measured at 180 min after completion of breakfast. (84). These findings align with other research, which indicates there is little evidence suggesting one source of protein is more effective than another as an aid for weight management (85–89).

## Isoflavones

Isoflavones, which are diphenolic molecules, naturally occur in plants and act by binding to both estrogen receptors (ER), ERα and ERβ, thereby influencing gene transcription.

The potency of isoflavones relative to estrogen is difficult to assess. Potency for compounds that bind to ERs is typically discussed in terms of relative binding affinity (RBA) and compared to 17β-estradiol, with the potency of the latter arbitrarily set at 100. Depending on the isoflavone and ER, estimates range from isoflavones being only about 1/1,000 as potent (90) to nearly as potent (91). However, RBA does not completely capture potency. The physiological effect of ligands binding to ERs will depend upon the conformational shape of the ligand-receptor complex, the relative ratio of the two ERs, and the types of co-repressors and co-activators in cells. All of these factors can strengthen or weaken the biological activity of the ligand. Also, there may be isoflavone metabolites formed within cells that are more or less potent than their parent compound (92, 93).

Isoflavones were identified as ER agonists in the 1950s (94, 95) and in the 1960s, as possible ER antagonists (anti-estrogens) (96). The main dietary source of isoflavones are legumes from the family Fabaceae (97), namely soybeans (*Glycine max*). Mean isoflavone intake in Japan among older adults ranges from ~30 to 50 mg/d (45, 98) whereas daily per capita intake in the United States (99–101) and Europe (102, 103) is <3 mg, although recent reports suggested daily intake may be as high as 7 mg in France (104) and was estimated at 4–6 mg among British adults although little of that came from soyfoods (103).

The three soybean isoflavone aglycones, genistein, daidzein, and glycitein, have molecular weights (g/mol) of 270, 254.2, and 284.3, respectively. These three aglycones and their glycosides (the predominant form in unfermented soy) account for about 50, 40, and 10%, respectively, of total isoflavone content (105). In plants isoflavones function as phytoalexins and as such accumulate during stress, such as during microbe attacks (106). Isoflavones also play a role in nitrogen fixation, thereby reducing the need for nitrogen fertilization (107, 108).

There is no precise estimate of the bioavailability of isoflavones although the European Food Safety Authority (EFSA) concluded it is low (109). In humans, there is a biphasic appearance of isoflavones in the plasma and urine following isoflavone ingestion. Isoflavones levels in the plasma occur 1–2 h, and then again at 4–8 h, following consumption (110–114).

Genistein inhibits the growth of a wide range of cancer cells *in vitro via* mechanisms unrelated to its ability to bind to ERs, although inhibition typically occurs at concentrations that are not achievable *in vivo* in response to intake within the dietary range (115). The demonstration that in comparison to ERα, soybean isoflavones preferentially bind to ERβ (91, 116), provides a molecular explanation for classifying isoflavones as selective estrogen receptor modulators (117). In general, activation of ERα and ERβ is seen as exerting proliferative and anti-proliferative effects, respectively (118).

Some of the proposed benefits of soyfoods, such as the promotion of bone health, alleviation of hot flashes and improvement of cognitive function, may be due to the estrogen-like effects of isoflavones. Finally, even if one accepts a low potency estimate for isoflavones relative to estrogen, it does not rule out possible physiological effects because blood levels of isoflavones in individuals consuming 30 to 100 mg/d exceed circulating estrogen levels in premenopausal women by many hundreds to 1,000-fold (119, 120).

## Fermented vs. unfermented soyfoods

Fermented soyfoods are frequently heralded over unfermented ones on social media because fermentation reduces the content of compounds that potentially inhibit nutrient absorption and the mistaken belief that Asians eat primarily fermented soyfoods. However, the clinical significance of this rather modest reduction is unclear. Furthermore, most soy consumed globally is unfermented, as fermented soyfoods play a small role in the cuisines of ethnic Chinese (Table 2) (45, 123). Worthy of note is that in the Shanghai Men's Health Study (SHMS), which comprehensively evaluated soy intake, the food frequency questionnaire included only questions about unfermented soyfoods because fermented soy intake is so low in Shanghai (124).

**Table 2:** Isoflavone intake from fermented and unfermented soyfoods in Japan, Korea, and China.

**References**	***N***	**Gender/age (years)**	**Location**	**Fermented**		**Unfermented**	
				**Foods (g/d)**	**Isoflavones (mg/d)**	**Foods (g/d)**	**Isoflavones (mg/d)**
Shirabe et al. (121)	11,983	F/45–74	Japan	Miso (20.2)	20.8 ± 17.8	Tofu (84.2)	24.2 ± 19.3
				Natto (27.6)		Soymilk (30.0)	
						Fried tofu (0.7)	
						Dried tofu (11.4)	
Lee and Kim (122)	4,025	M and F/≥20	Korea	Soybean paste	5.96	Soybean curd	4.55
				Dambuk	0.57	Soymilk	2.89
				Miso	0.17	Soy sprouts	2.08
				Seasoned soybean paste	0.14	Soybean	5.99
				Total	6.84	Soybean broth	0.72
						Total	16.23
Liu et al. (123)	1,188	F/22.7 ± 2.4	Rural China	Fermented tofu	0.3	Tofu	9.1
						Soymilk	0.1
						Sheet/slab	1.8
						Soymilk film	0.9
						Whole soybean	5.5
						Total	17.4
							

Shirabe: Data from 4th quartile isoflavone intake. Lee: National survey. Liu, data from Gansu and Hebei Provinces.

Whether fermentation affects isoflavone content is unclear, but it does affect isoflavone form. To varying degrees, fermentation converts isoflavone glycosides to aglycones. Murphy et al. (125) found that about one-third of the isoflavones in the fermented soyfoods miso and tempeh were in the aglycone form whereas in tofu, which is unfermented, typically <10% was in aglycone form. The degree to which this conversion occurs depends upon the bacteria used and the duration of fermentation (126, 127). Some studies have reported fermentation causes a decrease in total isoflavone content (128) whereas others have not (129). There are also conflicting data on the extent to which absorption is affected by the isoflavone form. Aglycones are absorbed more quickly, but total absorption may not be affected (114, 130, 131). The health implications of a faster absorption rate, and possibly higher peak circulating levels, are unclear.

Fermentation reduces protease inhibitor (PI) content (132), but its effect on protein digestion is unclear as the digestibility of protein from traditional unfermented soyfoods (52), soy protein ingredients (48) and foods made using these ingredients (53) is quite good. Older rat research suggests protein digestion is appreciably affected only when ~50% of the residual PI content remains (133). Fermentation also reduces phytate content, but its effect on mineral absorption is unclear (132). Phytate adversely impacts the absorption of calcium from soy (134); nevertheless, the absorption of calcium from calcium-set tofu (135) and calcium-fortified soymilk (136, 137) is similar to that of cow's milk.

Importantly, the results of single meal studies, which are typically used to determine bioavailability, may exaggerate the effect of enhancers and inhibitors of mineral absorption (138). Also, in contrast to older research (139), there may be adaptation to the inhibitory effects of phytate on iron absorption with chronic consumption of a high-phytate diet (140). Even so, the US iron RDA for vegetarians is 1.8-fold higher than for non-vegetarians because of the assumed lower bioavailability of non-heme iron in plant foods (141).

Observational studies tend to show tofu is more likely than miso to be associated with reduced risk of chronic disease (e.g., cardiovascular disease and various cancers), although it is difficult to control for all confounding variables that might be associated with possible differing patterns of use associated with these foods (142–145). And, some evidence indicates miso intake increases risk of developing gastric cancer (146, 147), although miso was recently found to be associated with an improved survival from this disease (148). Natto may benefit bone health (149–151) because of its high vitamin K content due to fermentation with *Bacillus subtilis* natto (152). Natto also contains *nattokinase*, an enzyme secreted by *Bacillus subtilis* natto (153), which exhibits fibrinolytic activity (154, 155). Furthermore, fermented soyfoods may function as probiotics, but this depends upon whether the product is pasteurized after the inoculum has been added (44, 156). Finally, fermentation has been shown to create antioxidants not present in unfermented soyfoods, but the clinical relevance of these molecules is unclear (157, 158).

**Perspective**: Overall, there appears to be little evidence that fermentation of soyfoods results in clinical benefit beyond that derived from unfermented soyfoods, but this issue has not been rigorously investigated. Natto is a notable exception because of its high vitamin K and *nattokinase* content. Data do not support general recommendations to choose fermented soyfoods over traditional soyfoods such as soymilk and tofu although fermented foods (tempeh, miso, natto) are based on the whole soybean whereas this is true of only some unfermented soyfoods (e.g., edamame, soynuts).

## Isoflavone-related topics

### Women with breast cancer (BCa) or at high risk of this disease

The historically low incidence rates of BCa in countries in which soyfoods have been a traditional part of the diet (159) helped fuel speculation that isoflavones exert anti-estrogenic effects thereby potentially offering protection against this disease (160). However, research published beginning in the late 1990s showed that genistein (28) and isoflavone-rich SPI (161) stimulated the growth of existing ER-positive mammary tumors in ovariectomized athymic mice. In addition, in this model isoflavones inhibited the efficacy of the breast cancer drugs tamoxifen (162, 163) and letrozole (164). These findings drew attention to the ER agonistic properties of isoflavones and led to clinicians advising their BCa patients to limit or avoid soy (165).

In contrast to the studies in mice, beginning in 1999 (166), clinical trials consistently showed that neither soy nor isoflavone consumption affected markers of BCa risk (167), including mammographic density (168–170) and *in vivo* breast cell proliferation (120, 166, 171–174) [Cells that proliferate more quickly are more likely to be transformed into cancer cells (175)]. These studies involved women with BCa, women at high risk of BCa and healthy women. In many cases, isoflavone intake greatly exceeded typical intake in Japan. In contrast to the lack of effect of isoflavones on cell proliferation, combined hormone therapy (CHT, estrogen plus progestin) increases proliferation (176, 177). CHT, although not estrogen alone, is known to increase risk of BCa (178). Since isoflavones do not possess progestin-like activity (179), the effect of CHT is potentially relevant to soy (179).

In 2009, the first prospective observational study to examine the impact of post-diagnosis soy intake on the prognosis of BCa patients was published (180). Among women participating in the Shanghai Breast Cancer Survival Study, post-diagnosis soy intake was associated with a significantly decreased risk of recurrence and BCa-specific mortality. Subsequently published observational studies conducted in the US (181, 182) and China (183, 184) aligned with these findings as was summarized by meta-analyses published in 2013 (185) and 2019 (186). Protective effects were observed in both ER-positive and ER-negative patients. No mechanisms for the protective effects have been proposed.

In 2012 [reaffirmed in 2021 (187)], the American Cancer Society (188) and the American Institute for Cancer Research (189); in 2014, the World Cancer Research Fund International (190), and in 2015, the Canadian Cancer Society (191), all concluded that women diagnosed with BCa can safely consume soy. However, the positions of these organization were based primarily on the epidemiologic data, not a comprehensive review of the literature. On the other hand, in 2015, the EFSA concluded isoflavone supplements (soyfoods were not examined) do not affect breast tissue in postmenopausal women (109). This conclusion was based on the animal, clinical and epidemiologic data. In 2018, the Permanent Senate Commission on Food Safety of the German Research Foundation (SKLM) reached a similar conclusion (192).

**Perspective**: The absence of clinical trials examining the impact of soy on BCa recurrence or mortality precludes claims that the soy and BCa controversy has been definitively resolved. However, given that clinical data are supportive of safety and the observational data are suggestive of benefit, the totality of the evidence is aligned with the positions of health agencies that women diagnosed with BCa can safely consume soyfoods. Although suggestive, the observational data do not provide a sufficient basis for recommending BCa patients begin consuming soy specifically to improve prognosis.

### Prostate cancer (PCa)

The historically low incidence rates of PCa in countries in which soyfoods have been a traditional part of the diet (193, 194) helped fuel speculation that isoflavones are protective against this disease, speculation which is biologically plausible since prostate tissue isoflavone concentrations exceed those in the blood (195). Several animal studies published in the 1990s provided support for a role of soy in PCa prevention. For example, in 1997, rats fed isoflavone-rich soy protein developed fewer chemically-induced prostate tumors than rats fed casein (196). Also, Zhou et al. (197) found soy protein plus isoflavones dose-dependently suppressed tumor formation in severe combined immune-deficient mice subcutaneously inoculated with prostate cancer cells. And, dietary genistein inhibited the progression of prostate tumors in a transgenic mouse model of PCa (198). Whether isoflavones exert protective effects in these models via the androgen receptor is unclear (199).

Several clinical trials also found that soy and isoflavone intake decreased prostate specific antigen (PSA) levels in men with PCa (200). PSA is a marker of prostate tumor growth (201). Of the eight trials identified in an older review involving men with PCa, four reported isoflavones slowed the rise in PSA levels and in four there was no effect (200). Although a few subsequently published studies found isoflavones or soy lowered PSA levels (202, 203), more recent work has not shown this to be the case (204, 205). The lack of efficacy is supported by a systematic review that included four studies, which were published in 2004, 2010, 2011, and 2013 (206).

Of the clinical trials that failed to show efficacy, two are especially notable because of their size and duration, One, which was stopped early at ~2 years because of a lack of efficacy, involved 177 men at high risk of recurrence after radical prostatectomy for PCa (204). Men were randomized to receive either 20 g/d SPI or casein. SPI provided 43 mg total isoflavones, of which 25 mg was genistein. Arguably, isoflavone exposure was low for a study examining PCa progression. In the other study, 300 men with confirmed high-grade prostatic intraepithelial neoplasia were randomized to receive daily a placebo or 40 g/d SPI (estimated isoflavone content, 100 mg) plus vitamin E and selenium for 3 years (205). The primary end point was time to development of invasive PCa. The lack of efficacy cannot be attributed to a low isoflavone dose, but the possible carcinogenic effects of vitamin E and selenium may have countered any protective effects of isoflavones (207, 208). However, there is also evidence that selenium reduces PCa risk (208).

Some observational evidence supports a protective effect of soy against PCa. For example, in 2018, a meta-analysis of 30 population studies found that both soyfood and soy protein intake were associated with a decreased PCa risk (2). However, the most robust findings were based on case-control, not prospective studies. The former carry less weight within the epidemiologic community. In contrast, another meta-analysis also published in 2018, which analyzed the combined results of the Japan Collaborative Cohort Study and the Japan Public Health Center-based prospective Study, found that serum genistein and daidzein concentrations were not significantly associated with PCa risk, although the odds ratios (ORs) for both isoflavones were below 1.0, which is suggestive of a protective effect (209). Finally, a population-based prospective study involving 43,580 Japanese men aged 45–74 years with no history of cancer found that over the median follow-up period of 16.9 years, isoflavone and soy intake was associated with a statistically significant *increase* in risk of PCa mortality (210).

**Perspective**: There is suggestive evidence that soyfoods reduce risk of developing PCa, but the data are too inconsistent to reach firm conclusions. Nevertheless, health professionals advising clients or patients concerned about developing PCa are justified in recommending that soyfoods be part of a dietary approach aimed at addressing this concern. Continued research is warranted but effects on PCa development or progression should not currently be a sole basis for recommending soy intake.

### Osteoporosis

The relationship between dietary protein intake and bone health is complex (211). Overall, studies suggest dietary protein has a neutral to possibly small beneficial effect on bone (212), but whether this depends upon the type of protein consumed is unclear (211, 213).

Early interest in the anti-osteoporotic effect of soyfoods stemmed from studies showing soy protein to be less hypercalciuric than animal protein (214, 215), an effect attributed to the lower SAA content (mg/g protein) of the former (216–218). However, the notion that animal protein causes bone dissolution as a result of its high SAA content (219, 220) has lost support (221–224), as has the hypothesis that soy protein improves calcium balance when compared to animal protein (225, 226). Nevertheless, the presence of isoflavones in soybeans continues to attract interest in the possible skeletal benefits of soyfoods because of the well-established skeletal benefits of estrogen (227).

Large prospective cohort studies from Shanghai (228) and Singapore (229), reported that soy intake was associated with a reduced fracture risk among women. In addition, a US prospective study involving Seventh-day Adventist (SDA) postmenopausal women, found soymilk intake was inversely related to risk of osteoporosis, although this may have resulted from calcium, rather than isoflavone intake (230). Interestingly, a recently published analysis of the SMHS found that high soy isoflavone intake (>45.2 mg/d vs. <21.7 mg/d) was associated with a significant reduction in risk of osteoporotic, but not non-osteoporotic, fracture (231). These findings conflict with the aforementioned Singaporean study which found soy intake was protective in women but not men. However, the Singaporean study did not sub-analyze the data according to fracture type (229). It is possible that the lack of effect in Singaporean men may have resulted from their lower isoflavone intake relative to men from Shanghai, although this lower intake did not prevent protective effects of isoflavones from being observed in Singaporean women.

In 1998, isoflavones were first shown clinically to improve bone mineral density (BMD) in postmenopausal women. (232). Over the past two decades clinical trials that examined markers of bone resorption and/or formation or BMD have produced inconsistent results. However, the authors of a meta-analysis of the clinical data that was published in 2021, reported a trend of isoflavones to increase bone formation markers such as bone alkaline phosphatase and osteocalcin (233). Additionally, there was a trend toward lower levels of pyridinoline and deoxypyridinoline, two bone resorption markers. In this analysis, ~1,000 women consuming a placebo were compared to over 1,000 women who consumed on average a daily dose of nearly 100 mg isoflavones. The trials ranged in duration from 3 to 24 months.

However, of the 4 large (*n* ≥ 100), long-term (≥2 y) trials that evaluated postmenopausal BMD (234–237), only one showed significant benefit (Table 3) (237, 238). This specific trial intervened with genistein in aglycone form and included osteopenic women (237) whereas the other three trials included healthy postmenopausal women and intervened with isoflavones in glycoside form. Finally, research published in 2015 (239), which involved the use of novel methodology to study bone loss in postmenopausal women found isoflavones to be efficacious and showed a more moderate isoflavone dose (~100 mg/d total isoflavones) increased bone calcium retention more than higher isoflavone doses (240). Two of the three aforementioned large, long-term trials showing no effect of isoflavones used daily doses of >100 mg (Table 3).

**Table 3:** Effects of isoflavones on bone mineral density in postmenopausal women in large, long-term clinical trials.

**First author, year**	**Location**	**Isoflavone dose (mg/d)^a^**	**Duration (years)**	**(*N*)**	**Effects on hip and spine BMD**
Marini et al. (237, 238)	Italy	54^b^	2,3^c^	389	Statistically significant increase
Alekel et al. (234)	USA	80 and 120	3	208	No statistically significant effects
Levis et al. (236)	USA	200	2	248	No statistically significant effects
Tai et al. (235)	Taiwan	300	2	416	No statistically significant effects

^a^Aglycone equivalent weight ^b^Genistein aglycone ^c^Osteopenic women, half of the initial participants continued for a third year. BMD, bone mineral density.

**Perspective**: The role of estrogen in bone health provides a theoretical basis for isoflavones to exert skeletal benefits, although isoflavones and estrogen differ at the molecular and clinical level [For example, unlike estrogen, isoflavones do not exert proliferative effects on endometrial tissue (241, 242) and as noted previously, isoflavones preferentially bind to ERβ (91, 116)]. The results of the observational and clinical data warrant additional research being conducted. However, only the results of sufficiently powered trials at least 2 years in duration hold the potential to meaningfully impact the current state of knowledge. At this point, it is premature to recommend isoflavone intake specifically for the purpose of improving bone health. Nevertheless, given that adequate protein is needed for bone health, isoflavones may have skeletal benefits, and some soyfoods and soy products are fortified with calcium, soyfoods can certainly be viewed as foods to emphasize for those concerned about bone health.

### Cognitive function

Results of the Honolulu-Asia Aging Study (HAAS) published in 2000 (26) raised concern that soy intake might impair cognition. This study found higher midlife tofu intake among men was associated with indicators of cognitive impairment and brain atrophy in late life. A *post-hoc* analysis using the men's intake as a surrogate showed the relationship between cognitive decline and tofu intake also applied to their spouses. It was theorized that soy isoflavones were acting as ER antagonists; at that time evidence suggested estrogen therapy might reduce the risk of developing dementia and Alzheimer's disease (243, 244). However, there were many limitations to this observational study, including that the study was designed to investigate coronary heart disease (CHD) not cognitive function and the dietary assessment included only 26 foods.

In contrast to the HAAS, the results of several small clinical trials published between 2001 and 2006 suggested that soy isoflavones provided primarily in the form of supplements exerted cognitive benefits (245–249), although this was not the case for a large 1-year study that intervened with 25 g/d SPI that provided 99 mg isoflavones (250). In 2008, the controversy that was started by the HAAS was reignited by an Indonesian cross-sectional involving older women and men that reported the consumption of tofu was associated with worse memory. In contrast, tempeh intake was associated with better memory, especially among those >68 years of age (31).

However, a follow-up study published in 2011 by the research group who conducted the cross-sectional study from Indonesia found positive linear associations of weekly tofu and tempeh consumption with immediate recall, which were significant in those with an average age of 67 years (251). Furthermore, in those with an average age of 80 years, the earlier reported negative association of tofu with immediate recall was no longer significant (251). More recently, a Japanese prospective observational study found higher midlife genistein intake was associated with cognitive impairment (252) although these results contrast with an earlier prospective observational study from Japan that found soyfood and isoflavone intake decreased risk of cognitive impairment in elderly women (253). They also contrast with the results of a large prospective Japanese study involving more than 40,000 adult men and women which found soyfood intake was unrelated to the development of disabling dementia over the approximate 20 year follow up period (254).

In 2014, a comprehensive examination of the animal, clinical and observation evidence found there was insufficient data to reach conclusions about the relationship between soy or isoflavone intake and cognitive function among older adults (255). However, in 2015, a meta-analysis of 10 placebo-controlled randomized trials that involved over 1,000 postmenopausal women, reported that isoflavones improved cognitive function and visual memory (256). Two years later, an analysis concluded that isoflavone supplementation improved executive function and memory domains of cognitively normal older adults in half of the studies evaluated (257). Finally, in 2020, a meta-analysis of 16 trials (1386 participants, mean age 60 years) found soy isoflavones improved memory and overall cognitive function (5). In the trials included in these meta-analyses, isoflavones were typically provided either in the form of supplements or an isoflavone-rich, concentrated source of soy protein. As to potential mechanisms for the observed benefits, a cross-over study involving older men and women reported that the consumption for 16 weeks of 67 g/d soynuts that provided ~25.5 g protein and 174 mg isoflavones, increased psychomotor speed performance, likely as a result of the increase in cerebral blood flow in 4 brain clusters, although executive function and memory were unaffected (258).

**Perspective**: The clinical data suggest isoflavones benefit cognitive function. Therefore, there is some evidence to recommend soyfood consumption as a means of delaying cognitive impairment. However, the totality of the evidence is too inconsistent to draw meaningful conclusions.

### Hot flash alleviation

Hot flashes are the most common menopause-related symptom experienced by women (259). A hot flash is a transient vasomotor event consisting of a sensation of warmth, typically accompanied by sweating, flushing, palpitations, and sometimes anxiety (260). Adlercreutz et al. (261) proposed in 1992 that isoflavones possess sufficient estrogen-like activity to mitigate the drop in circulating estrogen as women transition through menopause to alleviate hot flashes. Three years later the first clinical trial to evaluate this hypothesis was published (262). Over the years, many reviews and analyses of the results of the numerous soy/isoflavone-hot flash trials have been published but with contrasting conclusions. Most have suggested isoflavones are not efficacious (33, 263, 264) or offer at best modest benefits (265–269), whereas a smaller number have been more supportive of efficacy (270–274). Given that hot flashes are typically subjectively determined, there is a large and variable placebo effect (275), and since there are also large intra-individual differences in isoflavone metabolism (276), the inconsistent data are not completely unexpected.

However, in 2012, Taku et al. (4) offered an explanation for the inconsistency based on the differing genistein, but not total, isoflavone content of the supplements used in the clinical trials. A previously published narrative review had hinted at the importance of genistein content (272). In general, two types of soy isoflavone supplements have been used in clinical trials; one is derived from whole soybeans and has an isoflavone profile similar to that found in soybeans and soyfoods, that is, genistein is the predominant isoflavone. In contrast, the other type is made from the hypocotyledon (or germ) portion of the soybean and is quite low in genistein (~10% of total isoflavone content).

In the meta-analysis by Taku et al. (4), which included nine trials that evaluated hot flash severity (*n* = 988 women) and 13 trials (*n* = 1,196 women) that evaluated frequency, the net effect, that is the decrease in response to isoflavones minus the effect in the placebo group, was a 26.19% (*p* = 0.001) decrease in severity and a 20.62% (*p* = 0.00001) reduction in frequency. However, sub-analysis revealed that among studies that provided more than 18.8 mg/d genistein (median for all studies) the net reduction in frequency was 29.13% whereas it was only 12.47% among trials that intervened with supplements providing less than the median genistein intake (difference between groups, *p* = 0.03). One year later, a Cochrane review did not sub-analyze the data according to the isoflavone profile of the supplement, but did call for further investigation of the benefits of genistein for alleviating hot flashes (277). Clinical trials published after the meta-analysis by Taku et al. (4) are generally supportive of the efficacy of isoflavones (278–281).

**Perspective**: Clinical trials evaluating the efficacy of isoflavones to alleviate hot flashes, which date back nearly 30 years, have produced conflicting results, which are reflected in the reviews and analyses of the data published over this time. However, nearly all of these reviews have not considered the differing isoflavone profiles of the supplements used in the clinical trials. Trials that provide at least 20 mg genistein and at least 50–60 mg total isoflavones consistently show isoflavones to be efficacious for reducing the frequency and severity of hot flashes. Whether other endpoints which have been inconsistently affected in clinical trials involving isoflavones may be due in part to the differing genistein content of the supplement has not been examined.

### Thyroid function

Research of the effects of soy intake on thyroid function in rats was first published nearly 100 years ago (282). More relevant is the publication almost three decades later of several case reports describing goiter in infants fed soy infant formula (SIF) (283–285). However, the concern raised by these reports was allayed when the mineral iodine was added to the formula. In the 1990s, isoflavones were shown *in vitro* to serve as an alternate substrate to tyrosine for iodination (thereby potentially exacerbating thyroid function when iodine intake is marginal) and to inhibit the activity of thyroid peroxidase (TPO) *in vitro* and in rats (27). This enzyme is required for the production of both thyroxine (T4) and triiodothyronine (T3). In 2004, Conrad et al. (286) concluded that infants fed SIF had prolonged increases in thyroid stimulating hormone (TSH) levels based on a retrospective analysis of infants with congenital hypothyroidism. These increases were thought to be due to the inhibition of levothyroxine absorption by soy protein, not as a result of isoflavones exerting a systemic effect (287).

In 2015, the EFSA (109) and in 2018, the SKLM (192), concluded isoflavones do not affect thyroid function in postmenopausal women (the only group studied). In 2019, the first meta-analysis to examine the effect of soy and isoflavones on thyroid hormones, which included 18 clinical trials, found no effect on free levels of T4 or T3 (288). The analysis included trials that intervened with soy isoflavones, soy extracts, soy protein, daidzein-rich isoflavones or isolated genistein in doses ranging from 40 to 200 mg/d.

The above-referenced meta-analysis did find a very modest increase in TSH levels, although the authors of this work were unclear as to whether the increase was of clinical significance (288). TSH levels increased by only 0.248 mIU/L; normal reference values for TSH are 0.5 to 4.5 mIU/L. Furthermore, an examination of the forest plot from this paper shows that only four studies, all by the same research group, were responsible for the results showing an increase in TSH (289–292). Nevertheless, Tonstad et al. (293) found when comparing the 5th vs. 1st intake quintiles, there was an association between isoflavone and soy protein intake and elevated TSH levels (>5 mIU/l) among SDA women (*n* = 548), but not among men (*n* = 295). However, these findings are a bit surprising given the moderate intake; midpoint isoflavone and soy protein intakes among women in the 5th quintile were only 25.46 mg/d and 6.92 g/d, respectively. That is equivalent to approximately only one serving of a traditional soyfood daily.

As already noted, concern has been raised that soy intake may exacerbate thyroid function in individuals whose iodine intake is marginal. However, research published in 2012 involving 35 participants indicates this is unlikely because supplementation with 80 mg/d isoflavones for 3 months led to only negligible amounts of iodinated isoflavones (~0.01%) in urine samples (294). Finally, in subclinical hypothyroid patients, one cross-over study reported that 16 mg/d isoflavones provided by 30 g/d SPI for 8 weeks increased the likelihood of progressing from subclinical to overt hypothyroidism (291). However, a follow up study by this same research group in which participants consumed for 8 weeks the identical amount of SPI but that provided 66 mg/d isoflavones did not confirm these findings (295).

**Perspective**: Extensive evidence indicates isoflavones do not affect T4 or T3 levels in euthyroid individuals. There are conflicting data about the effects on TSH levels. Limited evidence indicates isoflavones are unlikely to impair thyroid function in individuals with subclinical hypothyroidism or whose iodine intake is marginal. Soy protein likely inhibits the absorption of levothyroxine, a drug used to treat hypothyroidism, but this is true for food in general and many dietary supplements, herbs and drugs (296). Hypothyroid patients do not have to avoid all soy as long as there is a sufficient time interval between soy consumption and levothyroxine ingestion. Recommendations are to consume the medication ~1 h before breakfast or to wait as long as fours after eating (297). Alternatively, as long as soy intake occurs in a consistent manner, the dose of levothyroxine can be adjusted appropriately so if necessary (298).

### Male hormones and fertility

The classification of isoflavones as phytoestrogens underlies theoretical concerns raised about effects on male fertility, which coincided with rising apprehension that environmental estrogens play a role in the declining sperm count occurring among men worldwide (299–301) and possibly contribute to the observed decline in testosterone levels (302, 303). Among US men, there has been a marked increase in testosterone testing, new initiation of testosterone administration, and even initiation of testosterone administration without recent testing, all of which is associated with exposure to televised direct-to-consumer advertising (304).

A few animal studies published around the turn of the century appeared to lend credence to concerns about soy. For example, in 1998, Strauss et al. (305) reported that genistein reduced serum and testicular testosterone concentrations and prostate weight in mice; in 2001, Weber et al. (306) found that an isoflavone-rich diet lowered testosterone levels in adult male Sprague-Dawley rats and in 2002, Sharpe et al. (307) observed that the neonatal feeding of SIF suppressed testosterone levels in marmosets.

More germane than the animal studies, is the publication of two case-control studies each describing single individuals who experienced feminizing effects [erectile dysfunction (29, 308), increased estrogen levels (29), loss of libido (29, 308), gynecomastia (29), low testosterone (308)] in response to excessive isoflavone intake (360 mg/d) and a pilot US case-control study which found an inverse association between soy intake and sperm concentration (but not count) among male partners of couples attending a fertility clinic (309). Sperm concentration was decreased largely because of an observed increase in ejaculate volume associated with soy intake, a finding that does not seem biologically plausible, especially considering that median genistein intake in the highest intake group was only 7.48 mg/d.

In contrast to these suggestive data, in 2021 a meta-analysis of 41 clinical trials conducted mostly in Western populations, found no effects of soy or isoflavones on reproductive hormone levels in men (310). Trials intervened with either isoflavone-rich soy protein or isoflavone supplements and involved men of all ages Total testosterone and free testosterone levels were measured in 1,753 and 752 men, respectively, and estradiol and estrone levels were measured in 1,000 and 239 men, respectively. Sub-analysis of the data according to isoflavone dose (<75 mg/d vs. ≥75 mg/d) and study duration ( ≤ 12 weeks vs. >12 weeks) also showed no effects. In addition to there being no effects on hormone levels, none of the three clinical trials to evaluate the impact of isoflavone intake on sperm or semen parameters showed any adverse effects (311–313), although one of these was not published in full manuscript form (313). Isoflavone doses ranged from 40 to 480 mg/d for durations from ~2 to 3 months.

In addition, neither of the two placebo-controlled clinical trials that evaluated the effects of isoflavones on breast tissue in men found evidence of gynecomastia. One of these studies, which involved 200 men, intervened with 66 mg/d isoflavones for 3 months (292) and the other ~100 mg/d for 3 years and involved >300 men (205). Finally, a study that included 184 men from couples undergoing *in vitro* fertilization, found that neither the intake of soyfoods nor isoflavones by the male partners was related to fertilization rates or a host of other fertility measures (314).

**Perspective**: Extensive clinical trial data show no effect of soy or isoflavones on testosterone or estrogen levels in men even when exposure markedly exceeds typical Japanese intake. More limited but consistent clinical evidence indicates no adverse effects of soy or isoflavones on sperm or semen parameters or risk of developing gynecomastia.

### Female hormone levels and menstrual cycle length

Concerns that isoflavones might affect circulating reproductive hormone levels in women, and in particular, raise estrogen levels, arose because isoflavones are classified as phytoestrogens. Isoflavones, can in theory, influence estrogen levels by virtue of effects on enzymes involved in steroid metabolism (315–318). They could also impact biologically active levels of hormones by affecting sex hormone binding globulin (SHBG) concentrations (319). However, evidence that isoflavones affect hormone levels in women is unimpressive, although via their interaction with ERs isoflavones can potentially affect biological processes affected by the hormone estrogen without affecting circulating estrogen concentrations.

A meta-analysis by Hooper et al. (320) published in 2009, found that based on 35 clinical trials involving postmenopausal women, that there were no effects of soy or isoflavone intake on estradiol, estrone, SHBG, follicle stimulating hormone (FSH) or luteinizing hormone (LH). In 11 studies involving premenopausal women, these interventions also had no effect on estradiol, estrone or SHBG concentrations. In contrast, FSH and LH levels were significantly reduced by about 20% based on seven studies (*n* = 73 women) using standardized mean differences (mean divided by the standard deviation of differences), but not mean differences. However, in sensitivity analysis when only studies at low risk of bias were retained, the results were no longer statistically significant. Furthermore, subsequent to this analysis, a 6-month study by Khan et al. (120), found no effect of isoflavones (235 mg/d) on FSH (LH was not examined) in 53 premenopausal women. Thus, the evidence does not suggest FSH is affected by isoflavones. In general, studies published subsequent to the meta-analysis by Hooper et al. (320) show a lack of effect of isoflavone exposure on hormone levels in women (8, 120, 236, 321–326).

Research on the impact of soy on menstrual cycle length (MCL) was first published by Cassidy et al. (327, 328) in 1994/95. The findings of this work led to concerns about infertility because MCL was increased as a result of a change in follicular phase length (~first 2 weeks of the menstrual cycle). However, ovulation was not prevented. In the meta-analysis by Hooper et al. (320), on the basis of 10 studies, soy/isoflavone intake was found to increase MCL by 1.05 d. Menstrual cycle function is suggested to be an indication of fertility (329, 330). However, *short*, but not long, menstrual cycles have been linked to 11–36% longer time to pregnancy (331–333).

No clinical research examining the impact of soy on MCL published subsequent to the meta- analysis by Hooper et al. (320) was identified. Limited older evidence suggested that longer cycles might be protective against the development of breast cancer (334). If the increase is due to an increase in follicular phase length as was noted for soy, in theory, women will spend less of their lifetime in the luteal phase, a period during which breast cells are more actively proliferating (335).

**Perspective**: Considerable clinical evidence indicates neither isoflavones nor soy impact circulating reproductive hormone concentrations in women. Less clear are the effects on FSH and LH. The impact of soy intake on MCL has not been studied for 15 years but MCL may be increased by soy by ~1 day, although ovulation is not prevented. The increased MCL is not expected to affect fecundity.

### Puberty onset

The effect of soy consumption on the onset of puberty has been the subject of limited investigation. This relationship is of interest in part because throughout the world pubertal characteristics are occurring at an earlier age (336–347); however, this trend is apparent in countries where soyfoods are part of the traditional diet as well as in countries that historically have not consumed soy (348). Two case-control studies found urinary isoflavone levels in Korean girls with precocious puberty were higher than in children without this condition (349, 350). However, there were several experimental design weaknesses to these studies (17) and the findings contrast with the results of a US cross-sectional study involving 327 SDA girls 12–18 years old that examined the impact of soy intake on age of menses onset (AOM) (351).

For this study, soy intake at their current age was used as a proxy for the soy intake of girls prior to the onset of menses. Neither total soy product intake nor the intake of soy-based meat alternatives, tofu/traditional soy, or soy beverages, was significantly related to AOM or the likelihood of early (<12 y) or late (≥14 y) AOM. (351). A similarly designed study involving 248 SDA boys age 12–18 y found (mean isoflavone, puberty onset) moderate (10.1 mg/d, 12.58 y) and high (54.9 mg/d, 12.50 y) isoflavone intake was significantly associated with earlier adjusted median age at pubarche (based on pubic hair development) in comparison to low-soy consumers (0.8 mg/d, 13.00 y) (352). However, in contrast, isoflavone intake was unrelated to a secondary measure of puberty, facial hair onset. Furthermore, in boys consuming the most soy, puberty onset was actually later than the average is for boys in the US (353).

Two small US clinical trials (354, 355) and one Japanese population-based cross-sectional study (356) examined the impact of soy intake on hormone levels in children. In the Japanese study, which involved 230 boys and 198 girls aged 3–6 y, after adjusting for potential confounding variables, higher soy intake was inversely related to urinary estrone and estradiol in boys and positively related to urinary testosterone and androstenediol in girls (356). Similar findings were reported for isoflavone intake. In contrast, no effects of isoflavone intake on hormone levels were noted in either of the two clinical intervention studies conducted. In one, estrogen levels were measured in 17 US girls aged 8–14 years, who consumed one serving of soy daily (average isoflavone intake, ~27 mg/d) for 8 weeks (354). The other study measured estrogen levels in eight girls and testosterone levels in four boys (aged 5–11 years) after consumption of a daily tablet containing16 mg or 48 mg isoflavones or a placebo for 8 weeks each in a randomized crossover design separated by 2 week washout periods (355).

**Perspective**: Limited evidence indicates there is no clear association between puberty onset and the intake of soyfoods. Additional observational and clinical research is warranted.

### Soy consumption during pregnancy

There are two issues to consider when addressing the impact of maternal soy consumption during pregnancy. One is the effect on the mother and the other the effect on the fetus. Neither issue has been examined extensively. Asian women consume soy during pregnancy as they do throughout other periods of life (357–359). For example, Miyake et al. (358) reported that the genistein and daidzein intake of 1,002 pregnant Japanese women participating in the Japan Osaka Maternal and Child Health Study was 15.0 ± 10.1 mg/d and 9.0 ± 6.1 mg/d (mean ± SD), respectively. These values are in alignment with those reported by Nagata et al. (360) (21.7 ± 13.7 mg/d), who also studied the total isoflavone intake of pregnant Japanese women (*n* = 194).

#### Maternal effects

Wang et al. (361) examined the association between the soy intake of pregnant women between 13 and 24 weeks of gestation in southwest China and risk of gestational diabetes mellitus (GDM) and cesarean section (CS). Participants in this prospective study were divided into the insufficient soy intake group (<40 g soy/d) and control group (≥40 g soy/d), as the latter is the amount recommended by the Chinese Nutrition Society. Among the 224 participants, there were 36 cases of GDM and 120 cases of CS. After adjustment, consumption of <40 g/d was associated with an increased risk of GDM (OR, 2.116; 95% confidence interval [CI]: 1.228, 7.907; *p* = 0.017), but not with CS.

These results align with the findings from another prospective Chinese study which involved 1,495 pregnant women, 529 of whom were diagnosed with GDM (362) At 6–14 gestational weeks, dietary information was collected by trained interviewers by 24-h dietary recall for 3 days including 2 weekdays and 1 weekend day. Mean soy intake was 8.7 ± 16.6 g/d (the assumption is that this value refers to g soy protein). When compared with non-soyfood consumers, the third soyfood intake tertile was associated with a decrease in risk of GDM (Relative Risk [RR] 0.73; 95% CI: 0.54, 0.99, *p* = 0.049).

Protective effects were also noted in a prospective Japanese study involving 84,948 women; during the follow up period, 1,904 developed GDM (363). After adjustment, compared with those in the lowest isoflavone intake quintile (mean, 8.4 mg/d), women in the highest quintile (mean, 64.0 mg/d) were significantly less likely to have GDM (RR, 0.82; 95% CI: 0.70, 0.95; *p* for trend = 0.05). Additionally, miso soup and natto, but not tofu intake, were inversely associated with GDM.

Possible clinical support for the observational data comes from a 6-week Iranian study involving 68 women with GDM who were randomly assigned to consume the control diet containing 0.8 g protein/kg body weight (70% animal and 30% plant protein) or a diet containing the same amount of protein but comprised of 35% animal protein, 35% textured soy protein containing 75 mg isoflavones and 30% other plant proteins (364). Compared to the those consuming the soy-containing diet, the control group had significantly higher fasting plasma glucose, serum insulin levels and the homeostasis model of assessment-insulin resistance. Somewhat parenthetically, the control group had a higher incidence of newborn hyperbilirubinemia (32.4% vs. 8.8%, *p* = 0.01) and newborn hospitalization rates (20.6% vs. 2.9%, *p* = 0.02).

Finally, Schiattarella et al. (365) recently concluded there is some evidence a vegetarian diet as well as a plant-based diet reduces risk of developing GDM and/or some symptoms of this condition. This conclusion aligns with recent work by Wang et al. (366), who found that among 2,099 Chinese women participating in the Tongji Maternal and Child Health Cohort, after adjusting for social-demographic characteristics and lifestyle factors, women in the highest quartile of plant-based dietary index (PDI) were less than half as likely to develop GDM. Soy products typically comprise a large part of the bean intake category, which represents a significant portion of the PDI.

**Perspective**: Intriguing although limited evidence indicates soy consumption during pregnancy reduces risk of developing GDM. Research aimed at better understanding this relationship is warranted.

#### Fetal effects

Despite the practice among Asians of consuming soy during pregnancy, concern has arisen that *in utero* isoflavone exposure could adversely impact the fetus (367, 368). In 2010, Balakrishnan et al. (369) demonstrated in an *ex-vivo* human placental perfusion model that genistein can transfer across the human placenta. Twenty years earlier, Adlercreutz et al. (370), reported maternal plasma isoflavone and cord and amniotic fluid values for seven Japanese women at delivery. Several other investigators have also provided data on isoflavone amniotic fluid and/or cord blood concentrations (360, 371–376). These data, which were recently reviewed by Messina et al. (17), led the authors to conclude that “.. in utero isoflavone concentrations are markedly lower than estrogen concentrations” and that because of this difference, “isoflavones are unlikely to exert an estrogenic effect on the fetus.”

Nevertheless, in 2000, a British prospective study, which included 7,928 boys born to mothers taking part in the Avon Longitudinal Study of Pregnancy and Childhood, found that mothers who drank soymilk [yes or no; OR, 3.67; 95% CI: 0.87, 15.44)] or who ate soy “meat” (≥1x/wk vs. never; OR, 2.95; 95% CI: 0.90, 9.68) during pregnancy were about 3-fold more likely to give birth to boys with hypospadias (a birth defect where the opening of the penis is on the underside of the organ) (377). However, these associations were not statistically significant (377). The authors speculated that isoflavones might be responsible for the apparent association; however, legume (dried peas, beans, lentils, and chick peas) intake was associated with a 7-fold increased risk of hypospadias (≥4x/wk vs. never; OR, 7.56; 95% CI: 2.25, 25.42), despite non-soy legumes containing negligible amounts of isoflavones (125, 378). Soy meat analogs, which typically contain very low levels of isoflavones, were also associated with an increased risk (125).

A large Japanese nationwide birth cohort study represents the most direct examination of the relationship between risk of hypospadias and isoflavone intake. Women were recruited for this study during early pregnancy (379).

The intake of genistein (median, 15.3 mg/d) was based on a self-administered food-frequency questionnaire. There were 51 cases of hypospadias among the more than 40,000 women who delivered singleton live male births. In comparison with mothers in the 11th−89th percentiles of genistein intake, those women in the low intake group ( ≤ 10th percentile) were nearly three times as likely to report having a son with hypospadias. In contrast, there was no relationship between the highest genistein intake (≥90th percentile) and risk of hypospadias. These findings led the authors to conclude that low isoflavone intake during early pregnancy may increase hypospadias risk. In addition to the genistein findings, low intake of tofu and natto, were each associated with about a 2-fold increase in risk.

Finally, Song et al. (380) reported that after adjusting for 10 potentially confounding variables, maternal intake during early pregnancy of several foods, one of which was soy, was associated with a reduced risk of ventricular septal defects (VSDs) in the offspring. The highest intake category was consumption ≥6x/wk. In China, where this study was conducted, VSDs occur in ~2.5 births out every 1,000.

**Perspective**: Only limited research has evaluated the impact of maternal soy intake on the fetus. According to one school of thought, *in utero* isoflavones concentrations are too low to an exert effects on the fetus. Older speculation that maternal isoflavone intake increases risk of hypospadias is contradicted by more recent research showing the opposite effect.

### Equol producers vs. equol non-producers

Equol was first isolated from equine urine in 1932 (381) and identified 50 years later in human urine (382). Twenty years later, it was proposed that those individuals whose large intestine host the microbiota capable of converting the isoflavone daidzein into equol, are more likely to benefit from soyfood consumption than those do not (383). Approximately 50% of Japanese fall into the equol-producing category, whereas only about 30% of Westerners do (384, 385). Equol is reported to be a more potent ER agonist than its precursor daidzein, providing a potential role for microbiota metabolism on the ultimate consequences of dietary exposure to daidzein (385).

So where does the equol hypothesis stand today, 20 years after it was first proposed? While it still remains to be proven, the hypothesis received support from a recently-published Japanese study that examined the relationship between isoflavone intake and the volume of white matter lesions among 91 cognitively normal elderly Japanese (386). Blood isoflavone and equol levels were analyzed ~6 to 9 years prior to the determination of white matter lesions. Circulating isoflavone levels were unrelated to the volume of white matter lesions; however, among the 23 study participants with the highest circulating equol levels, lesion volume was reduced by ~50%. White matter is found in the deeper tissues of the brain and contains nerve fibers that affect brain function and learning. White matter lesions disrupt brain function and are associated with an increased risk for cognitive impairment and Alzheimer's disease.

These Japanese results are biologically plausible because equol has been shown to reduce arterial stiffness, a significant determinant of white matter lesion volume in the elderly (387). The stiffer and harder the blood vessel walls, the more the heart must work to pump blood into the arteries. However, the impact on arterial stiffness cannot be the entire explanation, because clinical trials indicate that isoflavones (not just equol) also reduce arterial stiffness (388).

Since only a few clinical trials have directly administered equol to participants, the question that arises is whether equol is simply reflective of some unidentified phenotype or characteristic that leads to a different response to soy than the response of non-producers. While that is a distinct possibility, equol is biologically active in humans as clinical trials have shown equol alleviates menopausal symptoms (389). However, the same is also true for genistein, a soybean isoflavone which is not converted into equol (4).

Nothing about the equol hypothesis suggests that isoflavones do not exert beneficial effects in equol producers and in non-producers alike. Both equol and isoflavones can exert physiological effects. If equol does have benefits independent of isoflavones, a reasonable question is whether steps can be taken to convert non-producers into equol producers Some evidence indicates vegetarians are more likely to be equol producers than non-vegetarians (390, 391), which suggests diet potentially affects the intestinal bacteria in a way that can lead to equol production.

**Perspective**: The hypothesis proposed two decades ago that equol producers are more likely to benefit from soyfood consumption than non-producers remains intriguing, but unproven, and one that warrants continued investigation.

## Soy protein related topics

### Soy protein ingredients (concentrated sources of soy protein)

Much is known about the health effects of soy protein ingredients because these products are typically studied in animal and clinical trials, rather than traditional Asian soyfoods. For example, most information about soy protein quality (49, 50, 392–397), and its ability to lower cholesterol (398–405), and to promote gains in muscle mass and strength in response to RET (67) is based on studies involving SPI or SPC. Nevertheless, these concentrated sources of soy protein have raised concerns because of evidence suggesting they may increase levels of insulin-like growth factor-1 (IGF-1) (24) and for the extensive processing they undergo (19, 20).

Although there is a critical role for IGF-1 in normal growth and development, some evidence indicates elevated levels of this hormone may be a factor in the development of some cancers (406) and adversely impact longevity (407–410) On the other hand, higher IGF-1 levels have been linked with protection against cardiovascular disease (CVD) (411, 412). Additionally, higher levels of IGF-1 have been associated with a reduced risk of developing type 2 diabetes (413) in some studies although not all data concur (408). While some, but not all, evidence (414) indicates that soy protein may slightly increase IGF-1 concentrations, increases have been observed only at intake levels exceeding 25 g/d (415, 416). Other proteins, especially high-quality proteins, have also shown to increase IGF-1 (417), although there is some disagreement on this point (414).

As to the effects of processing, the manufacturing of SPI and SPC from soybeans results in a marked reduction in the fat and fiber content, as well as in most instances, an isoflavone concentration that is decreased by 80 to 90% (105, 418). Therefore, these ingredients should be viewed primarily as sources of protein. Ironically, recommendations to limit the intake of these products are sometimes made in an attempt to avoid excessive isoflavone intake (419).

Foods made using the soy protein ingredients, such as meat and dairy alternatives, are classified as group 4, ultra-processed foods (UPFs), according to the NOVA food classification system (420). However, a recent review concluded that the major criticisms of UPFs do not apply to these foods more so than to they do to their animal-based counterparts, meat and cow's milk, which are classified as group 1 foods or unprocessed/minimally processed foods (421).

Finally, as noted previously, the starting point for manufacturing concentrated sources of soy protein are soybean flakes, which are produced by crushing soybeans and removing the oil using a food grade solvent such as hexane. As such, claims have been made that residual hexane in products using these ingredients is a health risk (422). However, a review of residual levels of hexane in soy-based foods found “there is no evidence to substantiate any risk or danger to consumer health when foods containing trace residual concentrations of hexane are ingested.” (423). Also, it has been estimated that over a million soy burgers would need to be consumed daily before reaching hexane levels in rats shown to cause neurological problems (424).

**Perspective**: Nutritionists typically emphasize consuming whole foods, whether it be whole grains rather than refined grains, or whole fruit rather than fruit juice. This same approach can be applied to recommendations regarding foods based on soy protein ingredients. Nutritionists are justified in emphasizing the consumption of whole soyfoods (tempeh, edamame, soynuts) and minimally processed soyfoods (tofu, soymilk). However, foods based on concentrated sources of soy protein are convenient ways to obtain ample amounts of high-quality protein that for many people may be the only acceptable way to incorporate soy into the diet. The potential benefits and safety of concentrated sources of soy protein have been rigorously evaluated.

### Cholesterol reduction

The cholesterol-lowering effect of soy protein has been studied clinically for more than 50 years (425). A meta-analysis of the clinical data published in 1995 found, on the basis of 31 trials involving 564 participants, that soy protein reduced low-density-lipoprotein cholesterol (LDL-C) an estimated 12.9% (426). In 1999, after conducting its own analysis of the literature, the US Food and Drug Administration (FDA) approved a health claim for soyfoods and CHD (427). The FDA established 25 g/d as the threshold intake for cholesterol reduction. In contrast to the 1995 meta-analyses, more recent meta-analyses of the clinical data published between 2003 and 2019 show a range in LDL-C reduction to be a more modest level of between 3.2 and 6% (398–405).

The effect of soy protein is independent of the fatty acid content of soyfoods although the high PUFA content of traditional soyfoods represents a second mechanism by which incorporating soyfoods into the diet can potentially lower blood cholesterol (398). Soy protein may also lower blood triglyceride levels and slightly raise high-density-lipoprotein cholesterol levels, although the health claim is unrelated to these effects (399). Although no mechanism for the cholesterol-lowering effect of soy protein has been definitively identified, some authors have suggested that peptides formed from the digestion of soy protein upregulate hepatic LDL (428) and VLDL (429) receptors.

In 2007, the FDA announced its intention to reevaluate evidence in support of the health claim (34) and in 2017 (430), it announced its intention, pending public comment, to revoke the claim because the data were considered to no longer be sufficiently consistent to support an unqualified health claim (Unqualified health claims require significant scientific agreement). Of the 46 studies included in the FDA analysis, 19 (41%) reported that soy protein statistically significantly lowered LDL-C. While the data are inconsistent, they are no more so than they are for oat β-glucan (431) and phytosterols/stanols (432), both of which have unqualified CHD claims based on their cholesterol-lowering effects.

The FDA did not meta-analyze the results of the 46 studies it considered in its review. When this was done by Blanco Mejia et al. (405), soy protein was found to significantly lower LDL-C by 3.2%. Further, it was established via a cumulative meta-analysis, that at no time since the health claim was approved was the effect of soy protein on LDL-C not statistically significant (433). Like the FDA, in its review Health Canada also found a minority of studies (33%) reported a statistically significant reduction in LDL-C; however, it determined that most studies (81%) showed a reduction even if not statistically significant (404). Hence, it was concluded the direction of effect was consistent and for this reason, in 2015, Health Canada approved a cholesterol-lowering health claim for soy protein (432).

Finally, it is notable that as part of the process for evaluating efficacy, the FDA conducted a comprehensive safety review. In addition to examining the literature, the FDA addressed hundreds of comments submitted during the open comment period, many of which dealt with safety concerns. Although the FDA efficacy analysis focused on soy protein, most of the public concerns centered on isoflavones. These concerns were rejected as the FDA concluded that “the use of soy protein at the levels [25 g/d] necessary to justify a [health] claim has been demonstrated, to our satisfaction, to be safe…” (430).

**Perspective**: Soy protein has a modest, yet clinically relevant, cholesterol-lowering effect. The FDA is currently scheduled to make a final decision about the existing health claim in August of 2023. If this highest level claim is revoked, speculation is that it will be replaced with a strongly worded qualified health claim, such as the one that exists for soybean oil and CHD (434).

### Gout

Gout, the most common form of inflammatory arthritis worldwide, is caused by deposition of monosodium urate crystals in joints and various other tissues and appears in relation to chronic hyperuricemia (435). Estimates are that more than 9 million Americans have gout and more than 32 million have hyperuricemia (436). Worldwide an estimated 41 million people have gout (437). Age-standardized incidence rates of gout in South Asia, Southeast Asia, and East Asia, are moderately lower (~10%) than in Western Europe and North America (437). Gout and hyperuricemia can be considered components of metabolic syndrome, as insulin resistance leads to renal underexcretion of uric acid (438, 439). In the Third National Health and Nutrition Examination Survey (NHANES, 1988–1994), the prevalence of metabolic syndrome was 62.8% in patients with gout, compared with 25.4% in non-gout patients (440). Elevated uric acid levels may also increase risk of CVD (441).

A common perception among health professionals in Asia is that soyfoods increase risk of gout and potentially precipitate acute attacks in patients with this disease (361, 442, 443). For example, among the health professionals surveyed, 69, 46, and 27% in Singapore, Indonesia, and Thailand, respectively, consider consumption of soyfoods as a gout risk factor. This belief exists despite, with few exceptions, soyfoods not having an especially high purine content (444). To prevent gout, the Japanese Society of Gout and Nucleic Acid Metabolism recommends limiting purine intake to 400 mg/d (445).

However, the importance of patients with gout maintaining a low-purine diet has been deemphasized in recent years (446). A cross-sectional study involving >6,000 elderly participants with metabolic syndrome, found that non-soy legumes, despite being a purine-rich food, were inversely related to serum uric acid levels and the prevalence of hyperuricemia (447). One possible explanation for this lack of association is that serum uric acid levels are affected differently by purine bases and metabolites involved in the endogenous synthesis of purines (448). For this reason, dietary recommendations should be based more on how a food affects plasma urate, rather than on the purine content of a food (449).

Importantly, the results of intervention trials show that soy protein intake at levels as much as three times higher than the typical intake of older Japanese (~8–9 g/d) (45, 98) does not exert meaningful effects on blood uric acid levels (215, 450–457). It is noteworthy that the uric acid rising potential of soy purines (mainly adenosine and guanine) is much lower than those in meat and fish (higher proportion as hypoxanthine) (444, 448). This may be why a prospective study in gout patients reported that the impact of plant purine on gout attacks was substantially smaller than purine from animal sources (458).

Furthermore, population data suggest that soy intake may reduce risk of developing gout (459–462) and the guidelines of the British Society for Rheumatology for the management of gout include a recommendation to consume soybeans and other vegetable sources of protein (463); these guidelines align with the diet goals set forth by Beyl et al. (446) to consume tofu for the management of gout flares.

**Perspective**: Extensive clinical and limited observational data indicate that soyfoods do not increase risk of gout or appreciably affect serum uric levels. To the contrary, some recommendations call for increasing the consumption of soyfoods to manage gout. Aside from its effect on uric acid, soyfood intake may be advantageous for gout patients because some evidence suggests it favorably affects parameters of metabolic syndrome (464) and potentially decreases risk of CVD (465–467).

### Kidney stones

Kidney stones refer to the presence of renal calculi which results from an imbalance between the precipitation and solubility of salts in the kidneys and urinary tract. An analysis of data from 2013–2014 NHANES indicates that about 10% of Americans have a history of kidney stones (468). In most industrialized countries, ~80% of the kidney stones are composed of calcium salts, such as calcium oxalate (469). Risk factors for kidney stone formation include (1) higher body mass index (2) low fluid intake (3) low intake of calcium/low-fat dairy products (4) high intake of sugar sweetened beverages (5) low intake of fruits and vegetables (6) high sodium intake and (7) high animal protein intake (470). Dietary recommendations for at risk individuals (hyper-absorbers of oxalate, history of kidney stones), typically call for limiting oxalate intake to between 50 (471) and 100 mg/d (472). The Academy of Nutrition and Dietetics classifies foods containing >10 mg oxalate/serving as high-oxalate foods. As much as 50% of the oxalate in the urine comes from food when a typical diet containing 10 to 250 mg dietary oxalate is consumed, the other half coming from endogenous synthesis (473, 474).

Nevertheless, research shows a high oxalate diet is not associated with increased risk of kidney stones in the general population (475). Also, oxalate intake poorly reflects urinary oxalate levels (476, 477), likely because intestinal oxalate absorption and liver oxalate production varies among individuals (478, 479). Historically, patients were advised to decrease calcium intake to limit diet-dependent intestinal absorptive hypercalciuria (480, 481). However, prospective studies show low calcium intake increased risk of kidney stone formation (482–485). When calcium and oxalate are consumed together, a calcium-oxalate complex forms within the intestinal tract limiting the intestinal absorption and subsequent urinary excretion of free oxalate (481, 486).

Many soyfoods are relatively low in oxalate; one analysis found that of the 22 types of tofu examined only one contained >10 mg per serving, and both soymilks examined contained <6 mg/serving (487). Ellis and Lieb (488), reported values of ~5.3 mg and 4.0 mg per cup for two different soymilks. These values are much lower than high-oxalate foods such as spinach, rhubarb, peanuts, and chocolate, which contain >100 mg/100 g (489). However, several soyfoods contain >10 mg/serving including tempeh, soynuts and edamame; and some concentrated sources of soy protein are also quite high (487). It is difficult to generalize about the oxalate content of soyfoods because it varies among soyfoods and among different types of the same soyfood. Differences in oxalate values for a single food may be due to analytical methods, and/or biological variation from several sources, including cultivar, time of harvest, and growing conditions (489).

Of potential relevance is that soyfoods are high in phytate, a metal chelator. Absorbed phytate is excreted in the urine (490, 491) and may mitigate oxalate-induced kidney stone formation (483, 487). It is a strong inhibitor of calcium oxalate crystal formation *in vitro* (492) and in the Nurses' Health Study II, over an 8-year period women consuming the most phytate were 37% less likely to develop kidney stones (483). However, recent clinical work shows that despite phytate being excreted in the urine following supplementation, no changes in any of the well-established urinary risk factors for calcium renal stone formation were observed (493).

Massey et al. (494) found that oxalate absorption (mean ± SD) from soyfoods ranged from 2.1 ± 2.1% to 5.4 ± 4.2%, which is similar to other foods, and urinary oxalate excretion increased by 19.6 ± 23.3 to 124 ± 156 μmol (1.7 ± 2.1 to 10.9 ± 13.8 mg) during the 8 h following consumption of seven different soyfoods. They concluded that frequent soy product consumption may increase risk of kidney stones in susceptible individuals. However, normal urinary oxalate excretion is 110 to 440 μmol (10 to 39 mg) daily (494); therefore, it would appear that as long as other high-oxalate foods are avoided, soyfoods will not lead to hyperoxaluria.

**Perspective**: The oxalate content of soyfoods is likely not a concern for people not prone to developing kidney stones. For those who are, it is difficult to make recommendations about consumption because of the variation in the concentration of compounds in soyfoods potentially involved in the etiology of kidney stone formation including oxalate, protein, sodium, phytate and calcium. The decision to incorporate a given soyfood into the diet should be based on the specifics of that food placed within the context of the overall diet.

### Allergy

Food allergies are defined as “an adverse health effect arising from a specific immune response that occurs reproducibly on exposure to a given food.” (495). IgE-mediated food allergy is a significant public health issue that affects an estimated 3 to 10% of adults and 8% of children worldwide (496–499).

Although IgE from patients allergic to soy may bind to as many as 28 proteins from soy (500, 501), only eight (Gly m 1 to Gly m 8) have been registered by the International Union of Immunological Societies Allergen Nomenclature Sub-Committee (502, 503). β-conglycinin (Gly m 5) and glycinin (Gly m 6) are the major storage proteins as they account for about 70% of the whole soybean protein. These proteins are associated with severe allergic reactions in European soy-allergic individuals (504). Importantly, *Gly m* Bd 30 K, which is also known as P34, is viewed as the protein most likely to cause allergic reactions in soy-sensitive people (505).

Since over 200 foods have been shown to be allergenic (506), regulatory agencies have recognized the need to focus allergen labeling regulations on a limited set of priority allergens. In the US, soy is one of eight foods (Big 8) designated as a priority allergen that must be called out as an allergen on product labels when present in a food (507). The eight foods (milk, eggs, fish, shellfish, tree nuts, peanuts, wheat, and soybeans) requiring labeling are thought to account for 90% of food allergies among Americans. In Canada, the priority allergens are the same as in the US with the addition of sesame (508), which will officially soon be added to the US list (509). Somewhat parenthetically, the increased popularity of concentrated sources of pea protein is also resulting in more cases of allergic reactions to pea (510).

In Japan, seven food allergens, of which soy is not one, require mandatory labeling whereas in Europe, 14 foods, one of which is soy, require labeling (511). However, due to the lack of data on prevalence, severity and/or potency, or due to regional consumption of some foods, in 2021, the *ad hoc* Joint FAO/WHO Expert Consultation on Risk Assessment of Food Allergens recommended soy not be listed as a global priority allergen (512).

Five large surveys of food allergy prevalence in the US or Canada based on parent- or self-reported data, which likely overestimates prevalence, have been published over the past decade (498, 499, 513–515). These surveys highlight the variation in prevalence that exists among the major allergens. Among adults, soy allergy prevalence was reported to be 0.1, 0.16, 0.35 and 0.6%, which, in each case, made it the lowest prevalence of any of the major allergens. Among children and adolescents, these values were 0.25, 0.32 and 0.5%, which as in the case of adults, made soy the lowest prevalence.

Finally, estimates based on clinical experience, are that about 70% of children outgrow their soy allergy by age 10 years (516). Because highly refined soybean oil contains only negligible amounts of soy protein (517–522), soybean oil does not elicit allergic reactions in individuals sensitive to soy protein (523) and as such, does not fall under the allergy labeling regulation. In contrast, because it may contain small amounts of protein, this is not the case for lecithin derived from soybeans as it can potentially elicit allergic reactions (524–526). However, evidence suggests most soybean-allergic individuals do not react adversely to the ingestion of soybean lecithin.

**Perspective**: Soy protein is currently classified as a major allergen in the US although its prevalence is low relative to other major food allergens. Estimates are that about 3 out of every 1,000 adults and about 5 out of every 1,000 children, are allergic to soy protein. These individuals need to avoid all soy products containing protein but can consume highly refined soybean oil.

## Soybean oil/omega-6 fat and inflammation/oxidation

Soybean oil is the most widely consumed edible oil in the US (527) and the world (528). It accounts for over 7% of US caloric intake and over 40% of the intake of both essential fatty acids (529). Soybean oil is comprised of ~16% saturated fat, 22% monounsaturated fat and 62% PUFA (530). In 2017, the FDA approved a qualified health claim for soybean oil and reduced risk of CHD based on its ability to lower blood cholesterol levels when replacing saturated fat in the diet (434). In contrast to some vegetable oils high in n-6 fatty acids (e.g., corn oil, sesame oil), soybean oil contains considerable amounts of the essential n-3 fatty acid α-linolenic acid (ALA). A recent meta-analysis of prospective studies found ALA intake and tissue ALA concentrations were inversely related to all-cause mortality (531).

Despite the health claim, there are assertions that the intake of soybean oil, as well as other oils high in n-6 fatty acids, and that have a high n-6:n-3 fatty acid ratio, causes inflammation (21–23, 532) and that the US dietary n-6:n-3 ratio, of about 10:1 (529), is not compatible with optimal health. Some experts recommended dietary ratios as low as 2:1, (533) which is considerably lower than the ratio of ~7:1 in soybean oil. However, the notion that a higher n-6:n-3 ratio is harmful has been rejected by leading health agencies; instead, the emphasis is on making sure sufficient amounts of each type of fatty acid is consumed (534–542). Reviews of the clinical data show linoleic acid (LA) intake does not increase markers of inflammation (543, 544). This conclusion aligns with research showing that none of the seven clinical trials that evaluated the effects of soybean oil on inflammation found a statistically significant increase (545–551).

Beyond inflammation, there is concern that LA intake increases the oxidative susceptibility of LDL-C, thereby raising risk of CHD (21). PUFA are particularly susceptible to oxidation because of their multiple double bonds (552). The hypothesis that oxidized LDL-C promotes atherosclerosis was proposed more than 40 years ago (553, 554). Nevertheless, this hypothesis remains controversial (555, 556).

Furthermore, it is not clear that high-PUFA diets promote LDL-C oxidation more than high-saturated fat diets (557), although relative to PUFA, monounsaturated fat may increase LDL-C oxidation lag time (558). However, other dietary factors, such as antioxidants like vitamin E (559, 560), can greatly influence oxidation time, and likely have more impact than fatty acid intake (561). Of the three clinical trials (545, 562, 563) that examined the impact of soybean oil on oxidative markers, only one reported an increase (563). But in the context of the substantial LDL-C and small-dense LDL-C (sdLDL-C) lowering that occurred, and the *in vitro* assay used to assess time to sdLDL-C oxidation, the biological significance of this finding is unclear.

Finally, and most importantly, LA intake is not only associated with a decreased risk of CHD (564), but with diabetes (565), cancer and all-cause mortality (566). In addition, among participants of the European Prospective Investigation into Cancer and Nutrition, erythrocyte LA levels were inversely associated with risk of rheumatoid arthritis, an autoimmune and inflammatory disease (567).

**Perspective**: LA intake does not increase inflammation, a finding consistent with clinical trials showing soybean oil does not affect inflammation. In contrast, LA intake is associated with decreased risk of CHD and diabetes. Emphasis should be placed on making sure recommended intakes of both n-6 and n-3 fatty acids are met. When soybean oil replaces foods high in saturated fat, evidence indicates risk of CHD, and perhaps risk of other chronic diseases is reduced. The totality of the evidence indicates the susceptibility of high PUFA to oxidize is not a cause for concern. Although the data are more limited, research shows soybean oil ingestion does not increase oxidative stress.

## Intake recommendations

There are few if any soyfood intake recommendations from independent health organizations. As noted previously, the US FDA established 25 g/d soy protein as the threshold intake for cholesterol reduction (427). However, this threshold was established for regulatory (labeling) purposes and is not intended to suggest all hypercholesterolemic individuals should consume soyfoods. The Chinese Nutrition Society recommends pregnant women consume at least 40 g of soy daily, but this recommendation is made primarily because soy is an inexpensive means to increase protein intake in a region where meat is relatively expensive and intake is low (361). These conditions are not applicable to pregnant women in much of the developed world. He et al. (568) recently reported that the Chinese dietary guidelines call for consuming ≥10 g soybeans/1,000 kcal; however, in an older paper, Liu et al. (123) listed the Chinese dietary guidelines for soybeans as calling for the consumption of 50 g/d. In any event, it is not possible to determine from these Chinese recommendations an amount of soy protein or isoflavones to consume since, on a weight basis, soyfoods do not provide equal amounts of these components.

One basis for formulating an intake recommendation is to mimic the intake of regions that have traditionally consumed soyfoods, especially China and Japan, given their long history of consumption. Interestingly, national surveys in China indicate soy intake increased over a recent 40-year period (568) whereas in Japan, data indicate soy intake decreased, especially in relation to total protein intake (45, 569). Despite the increase in China, He et al. (568) reported that <30% of the Chinese population meets the ≥10 g soybeans/1,000 kcal recommendation. However, in comparison to Japan, dietary habits in China are much more heterogenous (123, 570). In some regions, relatively little soy is consumed whereas Shanghai likely represents the highest soyfood-consuming region in the world.

In the SMHS (*n* = 54,219) (571) and the Shanghai Women's Health Study (SWHS, *n* = 45,694) (572), mean (±SD) soy protein intake was 12.5 ± 7.94 g/d (~16.0% of total protein intake) (573) and 8.8 ± 6.3 g/d (13.4% of total protein intake), respectively. Mean (±SD) isoflavone intake (mg/d) in the SMHS and SWHS was 36.2 ± 24.4 and 40.8 ± 28.7, respectively. One analysis of the SWHS is particularly informative because data were provided on the extremes of intake. The mean isoflavone intake of the ~2.2% of the women who consumed ≥25 g/d soy protein was 145.7 mg/d whereas the mean isoflavone intake of the nearly 9% of the women who consumed <2.5 g/d soy protein was 7.4 mg/d (572).

In Japan, the mean second soy protein intake tertile was 9.8 g/d for male (*n* = 5,883) and 9.4 g/d for female (*n* = 7,638) participants of the Takayama prospective study (98). These values represent about 11% (men) and 13% (women) of total protein intake, assuming a total protein intake of 90 and 70 g/d for men and women, respectively, using intake values from the NIPPON DATA80/90 Nutrition Study (574) (Adult men and women in the US (575) and Europe (78) consume similar amounts of protein as Shanghainese and Japanese men and women). Mean isoflavone intake in the Japan Public Health Center–Based prospective study, which involved 83,064 men and women was 37.5 mg/d (576).

Importantly, because mean intake may not represent optimal intake, another approach for formulating intake recommendations is to consider the intakes associated with desirable health outcomes in observational studies. Although there is often a monotonic response, in most instances when statistically significant associations are found, the highest intake is associated with the largest protective effect. For example, in a recent analysis of the SMHS, the hazard ratios for osteoporotic fracture risk associated with isoflavone intake (mg/d) for quartiles 1 (reference, <21.7), 2 (21.7–32.1), 3 (32.2–45.2), and 4 (>45.1) were 1.00, 0.89, 0.91, and 0.73, respectively (231). Similarly, in the SWHS, there was a dose-response between soy protein intake and systolic and diastolic blood pressure, with the reduction being greatest in women consuming ≥25 g/d (572).

Finally, and perhaps most importantly, insight about intake can potentially be gained from the amount of soy protein and isoflavones that produce benefits in clinical trials. For the alleviation of menopause-related hot flashes, the previously referenced meta-analysis by Taku et al. (4) found that ~50 mg/d isoflavones is needed for efficacy. Studies included in a meta-analysis that found isoflavones improve flow mediated dilation (FMD) in postmenopausal women with low baseline FMD levels intervened with between ~50 and 100 mg/d (577). On the other hand, studies included in a meta-analysis involving mostly postmenopausal women that found isoflavones improve cognitive function intervened with between 60 and 160 mg/d (5).

In general, the clinical trials suggest the dose of isoflavones required for efficacy is greater than suggested by the observational studies. However, there are two important caveats regarding the interpretation of the clinical trials. One is that none of the individual studies included in the above-mentioned meta-analyses intervened with more than one isoflavone dose, and for the most part, the meta-analyses did not attempt to determine dose-response relationships. Two, although entirely speculative, it is possible that long-term consumption, as reflected in observational studies, can produce efficacious results in response to lower intakes than is needed for efficacy in relatively short-term intervention studies.

Based on the above discussion, a reasonable adult intake recommendation of 15–25 g/d soy protein and 50 to 100 mg/d isoflavones appears to be appropriate. Consuming amounts that exceed these recommendations is not associated with adverse effects, but there is little historical precedent for consuming more than these amounts. Also, given the dietetic principles of moderation and variation, and the benefit from consuming nutrients provided by other dietary sources of protein, it is reasonable to recommend that soy protein not account for more than ~25 to 30% of total protein intake. For average European (78) and American (575) men and women, this would be about 25 g/d soy protein.

## Author contributions

MM wrote the initial draft of the manuscript with contributions from JE, JK, VM, AD, and HL. All authors reviewed and commented on subsequent drafts of the manuscript and read and approved the final manuscript.

## Conflict of interest

MM was employed by the Soy Nutrition Institute Global, an organization that receives funding from the United Soybean Board (USB) and from industry members who are involved in the manufacture and/or sale of soyfoods and/or soybean components. JK was employed by Medifast Inc., a nutrition and weight-management company based in Baltimore, Maryland, that uses soy protein in many of its products. JE and AD are scientific advisors to the Soy Nutrition Institute Global. VM is married to MM, and employed by Nutrition Matters, which receives no funding from the soy industry. The remaining author declares that the research was conducted in the absence of any commercial or financial relationships that could be construed as a potential conflict of interest.

## Publisher's note

All claims expressed in this article are solely those of the authors and do not necessarily represent those of their affiliated organizations, or those of the publisher, the editors and the reviewers. Any product that may be evaluated in this article, or claim that may be made by its manufacturer, is not guaranteed or endorsed by the publisher.
